# Rotavirus vaccine will have an impact in Asia

**DOI:** 10.1371/journal.pmed.1002298

**Published:** 2017-05-09

**Authors:** Carl D. Kirkwood, A. Duncan Steele

**Affiliations:** Enteric and Diarrheal Diseases, Bill & Melinda Gates Foundation, Seattle, Washington, United States of America

## Abstract

Carl Kirkwood and Duncan Steele discuss the evidence supporting rotavirus vaccine deployment in Asian countries.

Diarrhea remains the second leading infectious cause of death among children under five years of age, with more than half a million deaths each year. Rotavirus disease accounts for 25%–30% of all severe diarrhea cases [1]. While every child is at risk of rotavirus infection, the vast majority of rotavirus deaths occur in low- and middle-income countries, particularly in sub-Saharan Africa and South Asia, where access to treatment for severe rotavirus-related diarrhea may be limited or absent. Rotavirus immunization is well recognized as the best approach to protect children from mortality and morbidity caused by severe rotavirus disease.

In 2009, the World Health Organization (WHO) recommended that all countries should include rotavirus vaccines in their national immunization programs, particularly those with high child mortality due to diarrhea [2]. Currently, 84 countries have introduced rotavirus vaccines into their national immunization programs, including 41 Gavi-eligible countries with financial support for vaccine procurement. The uptake of rotavirus vaccines in sub-Saharan Africa and the Americas has been excellent; however, progress in Asia has been insignificant, with a notable lack of introductions into national immunization programs despite the well-characterized burden of rotavirus disease [3,4]. Rotavirus disease and hospitalization have been significantly reduced in high- and middle-income countries, with multiple vaccine-effectiveness studies documenting their powerful impact [5]. Moreover, recent vaccine-effectiveness studies in low-middle–and low-income countries in Latin America and Africa have shown dramatic reductions in rotavirus-associated morbidity and mortality [6–8]. Thus, the large infant population at risk in Asia is a priority for future rotavirus introduction efforts.

The reasons for delayed vaccine introduction likely vary by country, with multiple stages along the pathway to implementation posing hurdles, including evidence gathering, decision-making, planning, and introduction. The driver for introduction may also differ; for example, perceived health benefits may be the primary reason in one area, and economic benefits may be more important in another. However, the limited data from low-resource populations across Asia, which are needed to provide evidence of the clinical protection that rotavirus vaccination provides against severe diarrhea, have also likely stalled the uptake of rotavirus vaccines within these regions.

In a recent study in *PLOS Medicine*, John Victor and colleagues describe effectiveness of the human monovalent rotavirus vaccine, (Rotarix) in Bangladesh [9], providing evidence that should help to change the status quo in the region. Victor and colleagues’ study is the first to evaluate protection in infants in a low-resource population in Asia, using the WHO-recommended schedule at 6 and 10 weeks of age (i.e., the visits corresponding to the first and second dose of diphtheria–pertussis–tetanus-containing vaccine [DPT 1 and DPT 2]). The trial used a cluster-randomized village approach, comparing Rotarix vaccination integrated into the routine childhood immunization program in Bangladesh to the standard childhood immunizations without rotavirus vaccine but still utilizing oral rehydration salt (ORS) and other routine standard of care. The vaccine reduced severe acute rotavirus diarrhea by 41.4% (95% CI 23.2–55.2) among vaccinees. However, vaccine-induced protection appeared to wane from 45.2% in the first year of life to 28.9% during the second year, with the latter estimate not reaching statistical significance. Also, this study did not identify any measurable indirect protective effects despite being designed to capture the full effects of a rotavirus vaccination program.

Interestingly, these effectiveness rates generated through the programmatic implementation of the vaccine are consistent with the Phase III efficacy results for another rotavirus vaccine, RotaTeq, in Bangladesh, which demonstrated 42.7% (95% CI 10.4–63.9) efficacy against moderate-to-severe rotavirus diarrhea [10]. The results also align with the Phase III efficacy data for Rotarix in Malawi: 49.4% (95% CI 19.2–68.3) [11]; waning protection was also noted in this clinical trial setting in the second year of life [12]. Finally, an indigenous Indian vaccine (Rotavac) recently demonstrated 53.6% efficacy (95% CI 35.0–66.9) against moderate-to-severe rotavirus diarrhea in India [13]. Thus, rotavirus vaccines implemented in Asia are likely to have a similar impact to that observed in Bangladesh in Victor and colleagues’ study and in Gavi-eligible countries previously.

Concerns about the costs associated with rotavirus vaccines showing limited efficacy have been raised. A recent examination of the cost effectiveness of rotavirus immunization in Bangladesh highlighted that the vaccine is cost effective, even in the scenario of no Gavi financing support (personal communication, C. Pecenka to C. Kirkwood). Similar health economic analyses consistently indicate that rotavirus vaccines are very cost-effective interventions for low- and middle-income countries with a high diarrhea burden [14].

With increasing regional evidence of the benefits of vaccination, the introduction of rotavirus vaccines in national immunization programs should be a priority for countries in the Asian region. In recent progress, India commenced introduction of locally manufactured vaccine (Rotavac), using a staged rollout that commenced in March, 2016. The first four states introduced the rotavirus vaccine into the state-based immunization program and included active monitoring for programmatic and safety concerns as the vaccine was rolled out. Vaccine effectiveness is also being assessed. During 2017–2018, the government plans to roll out the vaccine into an additional five states, reaching approximately 50% of the Indian birth cohort. Another large country, Pakistan, commenced routine rotavirus immunization, with Gavi support, in January 2017 and plans to expand immunization over the coming months. Finally, Gavi recently approved support for Bangladesh to introduce rotavirus vaccine, which is anticipated to launch in 2018.

Therefore, the report by Victor and colleagues is timely and provides excellent evidence for the health benefits of rotavirus vaccines within a low-resource setting in Asia. The vaccine-effectiveness data highlight that introduction in settings of high rotavirus disease burden will result in a large public health benefit through a significant reduction in morbidity and mortality associated with rotavirus infection. As India, Pakistan, Bangladesh, and other countries in the region scale up the programmatic use of rotavirus vaccines, we should see dramatic reductions in childhood mortality due to diarrheal disease. Furthermore, as many countries transition from Gavi support and subsequently have to pay the full vaccine costs, we will see the advent of new safe, efficacious, and lower-cost rotavirus vaccines from manufacturers in India and elsewhere in the region, which will support the long-term sustainability of national immunization programs.

## Abbreviations

CI: confidence interval
DPT: diphtheria–pertussis–tetanus vaccine
ORS: oral rehydration salt
WHO: the World Health Organization
